# Tracing spatial mid-size Eastern U.S. cities road dust pollution: insights from source apportionment and health risk assessment

**DOI:** 10.1007/s11356-026-38034-x

**Published:** 2026-07-07

**Authors:** Minh Tri Truong, Chattan T. Haselden, Anh-Chi Tuan, Justin B. Richardson

**Affiliations:** 1 Department of Environmental Sciences, University of Virginia, Charlottesville, VA 22903 USA; 2 Department of Mathematics, Politecnico di Milano, Milan, Italy

**Keywords:** Road dust pollution, Potential toxic elements, Source apportionment, Health risk assessment, Geospatial analysis, Dimensionality reduction

## Abstract

As urban areas expand in eastern USA, the convergence of historical and modern anthropogenic source inputs has resulted in a complex geochemical signature of road dust pollution, while representing a critical public health issue for communities. In this study, road dust collected at seven (7) cities in eastern USA was analyzed for 11 potential toxic elements (PTEs, e.g., Cu, Zn, As, Se, Ni, Fe, Mo, V, Co, Cd, Pb) and examined for their characteristics, sources, and potential health risks. Multivariate statistical analyses show the regional difference between northeastern (Trenton, NJ; Wilmington, DE), Piedmont (Richmond, VA; Raleigh, NC; Greensboro, NC), and southeastern cities (Charleston, SC; Augusta, GA; outlining the spatial variability of eastern USA  road dust sources. Above-unit enrichment factors (EFs > 1) from Cu, Zn, Mo, and Ni imply accumulation from non-natural sources, such as non-exhaust traffic emissions and industrial activities. Hazardous PTEs (e.g., Pb, As, Cd) exhibited EFs < 1, reflecting their historical input in surveyed cities, and were associated with low-income communities. Source apportionment approaches estimate a one-third contribution from hazardous PTEs (coal combustion, insecticide use) and two-thirds from other prominent urban sources (waste incinerators, vehicle emissions, and industrial activities). Trenton, Raleigh, and Greensboro also see a higher respirable dust fraction (< 10 µm) than other cities, leading to potentially higher inhalation health risk. Hazard index (HI) estimation shows overall 4.5-times higher values in children than adults across all cities, with Augusta, GA exhibiting elevated hazard exposure (HI > 1). Insights from this study revealed the spatial variability of road dust PTEs levels, complemented domestic legacy contaminant work, and revealed new source information for residential areas over the East Coast to highlight potential environmental impacts.

**Supplementary Information:**

The online version contains supplementary material available at 10.1007/s11356-026-38034-x.

## Introduction

Roadside dust particles could form by weathering processes of inorganic and organic materials and are easily dispersed via dry deposition (Khan and Strand 2018). A variety of processes control road dust physiochemical properties, including dispersion, resuspension, and chemical transport (Dietrich et al. 2022). Therefore, road dust accumulation contains inputs from multiple sources, increasing exposure risk of potential hazards for public health yet remains an underutilized medium for scientific studies (Tong et al. 2023; Gokey et al. 2025). Despite being a natural pollutant sink, road dust in its finer size fractions is susceptible to resuspension, becoming a wind-induced threat that spikes local pollution hotspots (Coburn, 2005; Linda et al. 2025). Emergent, mid-size cities (generally > 200,000 residents) become the ideal target for dust pollution studies, where skewed development might lead to imbalanced exposure of pollutants among residents **(**McFarland et al. 2022; Jones et al. 2022; Daniel et al. 2024**)**. The influx of new population from gentrification on top of historically industrial cities proposed new sources and pollution exposure besides legacy contaminants, creating a mixed scenario necessitating proper pollution and health risk assessment (Wang et al. 2022; Wade et al. 2021; McFarland et al. 2022).

Road dust composition consists of an array of elements, to each their own reflect their natural and modern inputs (e.g., industrial, anthropogenic). A few hazardous elements are historically tied with cities’ industrial development and migration (e.g., Pb, As, Cd) that are more greatly exposed to residents in older cities, spiking up their toxic heavy metals’ levels in blood, urine, and hair (Obeng-Gyasi et al. 2021; Thorstenson et al. 2025). A geochemical and geospatial framework, therefore, remains critical in addressing the pollution hotspots, impacted regions and risk assessment of PTEs in road dust matrix. Dietrich et al. (2022) outlined Pb, Cu, and Cr as most reported PTEs in road dust studies, indicating the influence of non-exhaust vehicle emission (e.g., break wear and tire wear) and Pb contamination in nationwide studies. Notably, Pb contaminant legacy has been documented widespread, with a recent U.S. EPA guideline level revision to 200 mg kg^−1^ at residential CERCLA (Superfund) sites, hinting a change in approach of pollution remediation and stricter regulations in urbanized scenarios (Fillippelli et al. 2024, Saleh et al. 2025, USEPA 2025). However, current PTEs screening guidelines are mostly advisory from assumptions of redox states and speciation when only bulk concentration is reported, while PTEs threshold for road dust pollution has yet to be established.

Eastern U.S. municipalities are bouncing back to a net 1.0% increase in population, showing an expansion of residential areas in recent years. However, little data was reported for air and dust pollution studies in these regions, especially in the southeastern states (USEPA, 2015). While northeastern states are known for their Superfund program (e.g., NJ, NY and PA); southeastern U.S. states were known for their industrial parks, extended mining operations (e.g., NC, SC), and agricultural contribution to environmental pollution (Etteieb et al. 2020; Kiaghadi et al. 2021). Nonetheless, Virginia and the Carolinas are located on the Piedmont Plateau, with possible mixing of historically insecticide use from decades of cotton, tobacco farming, and urbanization (Richter et al. 2015; Schroeder et al. 2022). This created a mixed set of contamination sources to road dust, but with spatial heterogeneity between cities that calls for a proper assessment methodology. Existing studies have successfully characterized local PTEs pollution sources and outlined their related risks but remained limited to one city without spatial analysis and the regional context of potential implication to urban population (Fussel et al. 2022; Liu et al. 2023; Kasongo et al. 2024; Roy et al. 2024; Liu et al. 2025; Lee et al. 2025).

Nevertheless, chronic exposure of toxic PTEs is commonly associated with low-income communities, with closer proximity to traffic sources, waste incinerators, and risk management plan facilities (Jones et al. 2022; Aguila-Gomez et al. 2025). For example, elevated Cd, Cr, Cu, Pb, Zn, and Hg concentrations in road dust are known carcinogens that might cause adverse health effects upon long-term exposure (Dietrich et al. 2022). Notable adverse effects included those on skeletal, respiratory, and reproductive system from overconsumption of lead (Pb), cadmium (Cd), and arsenic (As; Tchounwou et al. 2012), which are mostly higher near former emission sources (e.g., smelter, incinerators) than residential areas, which could lead to certain social implications from road dust pollution (Lusby et al. 2015; O’Shea et al. 2021). Notably, median household income (MHI) often serves as a proxy for exposure as these communities live in smaller homes with older infrastructures, higher population density, and disproportionately located on legacy industrial land uses (Klepeis et al. 2017). Despite a well-document body of literature in mega-municipalities like New York City, NY and Philadelphia, PA; more data report from mid-size eastern U.S. cities would help expand the framework, especially in these cities where a great degree of gentrification spiked up concerns for population dynamics (Cole and Foster 2001; Corburn 2005). Therefore, the objectives of the study are as follows:

- Report the 11 PTEs abundance (Cu, Zn, As, Se, Ni, Fe, Mo, V, Co, Cd, Pb), their enrichment ratios and spatial variability at 7 mid-size eastern U.S. cities in the context of domestic and regional road dust pollution.
- Outline PTEs urban sources using multivariate statistical tools (principal component analysis, dimensionality reduction, correlation analysis) and source-receptor modelling (positive matrix factorization) to assess the regional input of common anthropogenic and industrial sources (combustion, waste incineration, traffic, etc.)
- Apply a geospatial toolbox to outline PTEs hotspots and overlay with MHI to assess their impacts. Also, assess the road dust grain size distribution and health risk assessment for insights of PTEs exposure disparity in surveyed cities.

This study seeks to complement existing research on road dust and topsoil pollution throughout the conterminous USA, utilize a geochemical and geospatial approach that focuses on eastern mid-size cities, and compare/contrast PTE contaminants that may potentially impact human exposure and health risk assessment in these areas.

## Methodology

### Sampling sites and sample collection

**Fig. 1:**
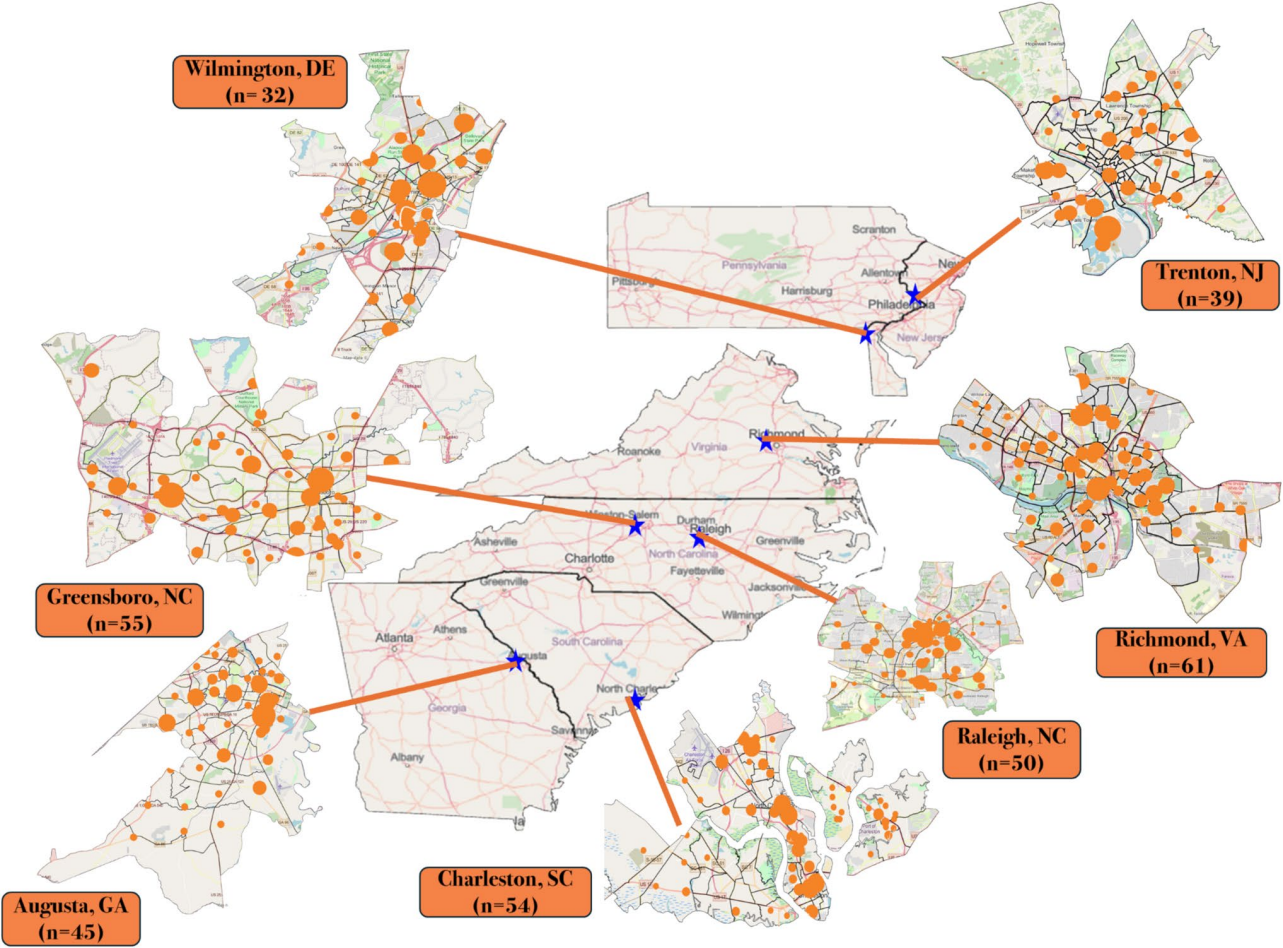
Sampling cities in this study

In this study, road dust was collected in August 2025 in the cities of Trenton, NJ (*n* = 39); Wilmington, DE (*n* = 32); Richmond, VA (*n* = 61); Greensboro, NC (*n* = 55); Raleigh, NC (*n* = 50); Augusta, GA (*n* = 45); and Charleston, SC (*n* = 54). The chosen spatial variability covers a wide array of industrial legacy, city development, and climate variability, with the bonus advantage of mid-size cities (71,000–299,000 residents) for traffic influence and heterogenous PTEs exposure. Road dust was collected by sweeping a normalized area of 1 × 1 m^2^ using a broom and dustpan into scintillation vials. Samples were collected in public and open spaces (i.e., residential streets, public parks, and schoolyards) that are in the closest possible proximity to a street to investigate possible contributions of traffic activity. Road dust near potential point sources (i.e., known industrial areas, railroads) were also collected for investigation. To avoid possible dust scavenging by precipitation, a 48-h no-rain window was guaranteed before sample collection. Collected samples were then labeled, digitally recorded using ArcGIS Survey123, and transported back to Soil Biogeochemistry lab at Department of Environmental Sciences, University of Virginia for storage and analysis. The map of sampling sites is shown in Fig. 1.

### Sample processing and data analysis

#### ICP-OES, ICP-MS analysis and data visualization

Road dust samples were air dried then sieved through a 250-µm mesh to better examine the inhalable (> 100 µm) and respirable (< 10 µm) dust fractions. Sample digestion followed a revised EPA Method 3050B protocol, in which ~ 0.5 g portion were mixed with a 9:1 (v/v) mixture of HNO_3_:HCl at 80 °C for 1 h, then diluted with 18.2 MΩ deionized water before analysis (Rice et al. 2024; Kohlhaas et al. 2026). PTEs concentrations were then quantified by Agilent 5800 ICP-OES and Agilent 7900 × ICP-MS instrument (Santa Ana, CA, USA). Periodical QA/QC flags including analytical blanks, duplicate samples, and SRM recovery checks using SRM 2710a (Montana I soil) and SRM 2586 (trace element in leaded soil) were employed for method validation and data cleaning and curation. All SRM samples yielded 80–120% recovery rates for certified PTEs in both methods, ensuring methodological capability (Table S1). Data analysis and visualization were performed using Python (v3.12), with Pandas and Numpy for data processing; Scipy, Scikit-learn for statistical analyses and Matplotlib, Seaborn for figures generation (Hunter 2007; Pedregosa et al. 2011; Harris et al.^2020^; Waskom 2021).

#### Enrichment factor

Enrichment factor (EF) measures the degree of enrichment between samples (i.e., presumably contaminated matrix, e.g., road dust) as compared to background (i.e., subsurface soil). Strontium (Sr) is used as the background normalizer for its abundance in crustal layer (0.2–20 mg kg^−1^, ATSDR, 2004) and minimal input from non-natural processes, while being resistant to weathering from redox and leaching processes (Carling et al. 2020; Marcy et al. 2024; Munroe et al. 2025). The choice of Sr as a normalizer overcomes conventional elements like Fe or Al, minimizing normalization bias in urban dust matrices (Fiala and Hwang 2021; Denny et al. 2022):

1 $$EF=\frac{{\left(\frac{{C}_{i}}{{C}_{Sr}}\right)}_{\mathrm{s}\mathrm{a}\mathrm{m}\mathrm{p}\mathrm{l}\mathrm{e}}}{{\left(\frac{{C}_{i}}{{C}_{Sr}}\right)}_{\mathrm{b}\mathrm{a}\mathrm{c}\mathrm{k}\mathrm{g}\mathrm{r}\mathrm{o}\mathrm{u}\mathrm{n}\mathrm{d}}}$$

where C_i_ is the concentration of a given PTE of interest and C_Sr_ is the concentration of Sr in a road dust (sample) and subsurface soil (background).

### Geospatial kriging and bivariate analysis

Spatial hotspot analysis was conducted using empirical Bayesian kriging in the geostatistical toolset in ArcGIS Pro (v 3.4, Redlands, CA, USA). The parameters for the analysis were adapted from McLagan et al. (2019) and listed in Table S2. Emission hotspots were projected by performing log-transformed concentration interpolation for selected PTEs, using Jenks natural break classification in 5 classes for optimal display. Resulted raster are then clipped in city layers composed of 2020 U.S. Census Tracts that covering sampling points in each city. The color palette was not normalized on the same scale for all cities, and color legends were excluded to emphasize true concentration numbers from data points. To illustrate the potential impact of skewed PTE exposure, a “low-income” threshold of < 80% area median income (AMI) was referenced from Fannie Mea’s area median lookup tool to avoid city-to-city bias (Fannie, n.d.). This criterion is widely applied in U.S. mortgage policy to determine low-income households to benefit for affordable housing.

### Data dimensionality reduction and source apportionment

#### Sample dimensionality reduction and projection

Multivariate dimension reduction tools including t-distributed Stochastic Neighboring Embedding (t-SNE) and Uniform Manifold Approximation Projection (UMAP) were chosen to better assess the primary drivers in PTE concentration variability between cities. Kruskal–Wallis tests were employed to test if there were significant statistical differences between cities for each PTE while eta-squared (η^2^) analysis ranked the elements from most to least discriminatory power. A perplexity value of 15 was used for t-SNE for better projection result, focusing on locally tight clusters. The number of nearest neighbors and maximum distance (n_neighbor = 100 and min_dist = 0.005) was optimized for UMAP to maximize the local structure while maintaining a reasonable global structure. t-SNE and UMAP have been commonly used to visualize grouping patterns for complex environmental datasets, with a recent focus on environmental pollutants (Liu et al. 2021; Badawy et al. 2025).

#### Pearson’s correlation, PCA and PMF analysis

Pearson’s correlation and principal component analysis (PCA) on log-transformed, normalized PTEs concentration were performed by scikit-learn and scipy Python packages (v 3.12, Pedregosa et al., 2011). Varimax rotation was included in maximizing PTEs’ variation in the two first principal component (PCs). Besides, a U.S. EPA-built model, positive-matrix factorization (PMF) seeks to quantify the contribution of sources outlined by PCA. PMF calculates the contribution and uncertainties by decomposing each PTE into a factor profile (f) and factor contribution (g). Further details regarding PMF analysis framework can be referenced to Truong et al. (2022)**.**

### Road dust grain size analysis

Representative sieved road dust samples (< 250 µm) with high PTE levels compared to U.S. EPA (200 mg kg^−1^ for Pb, 0.68 mg kg^−1^ for As) were analyzed for grain size distribution using a BetterSizer S3 Plus Laser Diffraction Particle Size and Shape Analyzer (Costa Mesa, CA, USA) at the University of Virginia. Roughly 2 g of road dust was dispersed in 0.05% sodium hexametaphosphate overnight (i.e., 12 h), then pipetted into the sampling bath for analysis. Size fraction abundance within the 0.01–3500 µm range was captured by dual camera lens and laser diffraction during analysis, and data was visualized using Python (v3.12). Due to the limited sample size, selected samples are analyzed to grain size distribution and elemental concentration for each size bin is not available per requirements of EPA Method 3050B.

### Health risk assessment

Five PTEs (Pb, As, Cd, Cu, Zn, with known toxicity to human health) were selected for health risk assessment via three exposure pathways: ingestion, inhalation, and dermatological pathways. Average daily doses (ADD_i_, in mg kg^−1^ day^−1^) derived from measured PTE concentrations were compared to EPA reference does (RfD_i_, in mg kg^−1^ day^−1^) to derive hazard quotient (HQ) and hazard index (HI), using Eqs. (2–6).

2 $${ADD}_{ing }= \frac{C\times {IR}_{ing}}{BW}\times \frac{EF \times ED}{AT}\times CF$$

3 $${ADD}_{inh }= C\times {IR}_{inh}\times \frac{EF \times ED}{BW\times {AT}_{n}}\times CF$$

4 $${ADD}_{derm}=\frac{C\times SA\times AF\times ABS}{BW}\times\frac{EF\times ED}{{AT}_n}\times CF$$

5 $$HQ=\frac{{ADD}_{i}}{{RfD}_{i}}$$

6 $$HI=\sum {HQ}_{i}={HQ}_{ing}+{HQ}_{inh}+{HQ}_{derm}$$

in which exposure frequency (EF), exposure duration (ED), average time (AT), conversion factor (CF), ingestion rate (IR_ing_), inhalation rate (IR_inh_), dermal surface exposure area (SA), adherence factor (AF), and dermal absorption factor (ABF) were referenced from the U.S. EPA regulation outline (USEPA, 2015; 2017; 2025). Children (< 13 years old) and adults (13–65 years old) body weight (BW) were referenced from U.S. CDC Health Statistics in 2025 (Fryar et al. 2025). A HI > 1 indicates concerning HM exposure for the chosen population target. However, the HI assessment fell short in addressing the seasonal coverage as we attempted to address the spatial pattern and underlining the skewed exposure of PTEs in eastern U.S. mid-size cities. An indirect association between road dust particle size distribution and actual inhalation and ingestion exposure also plays as a limiting factor for residential health implication assessment.

## Results and discussions

### Road dust PTEs characteristics

#### Spatial variability of PTEs road dust concentration

**Fig. 2:**
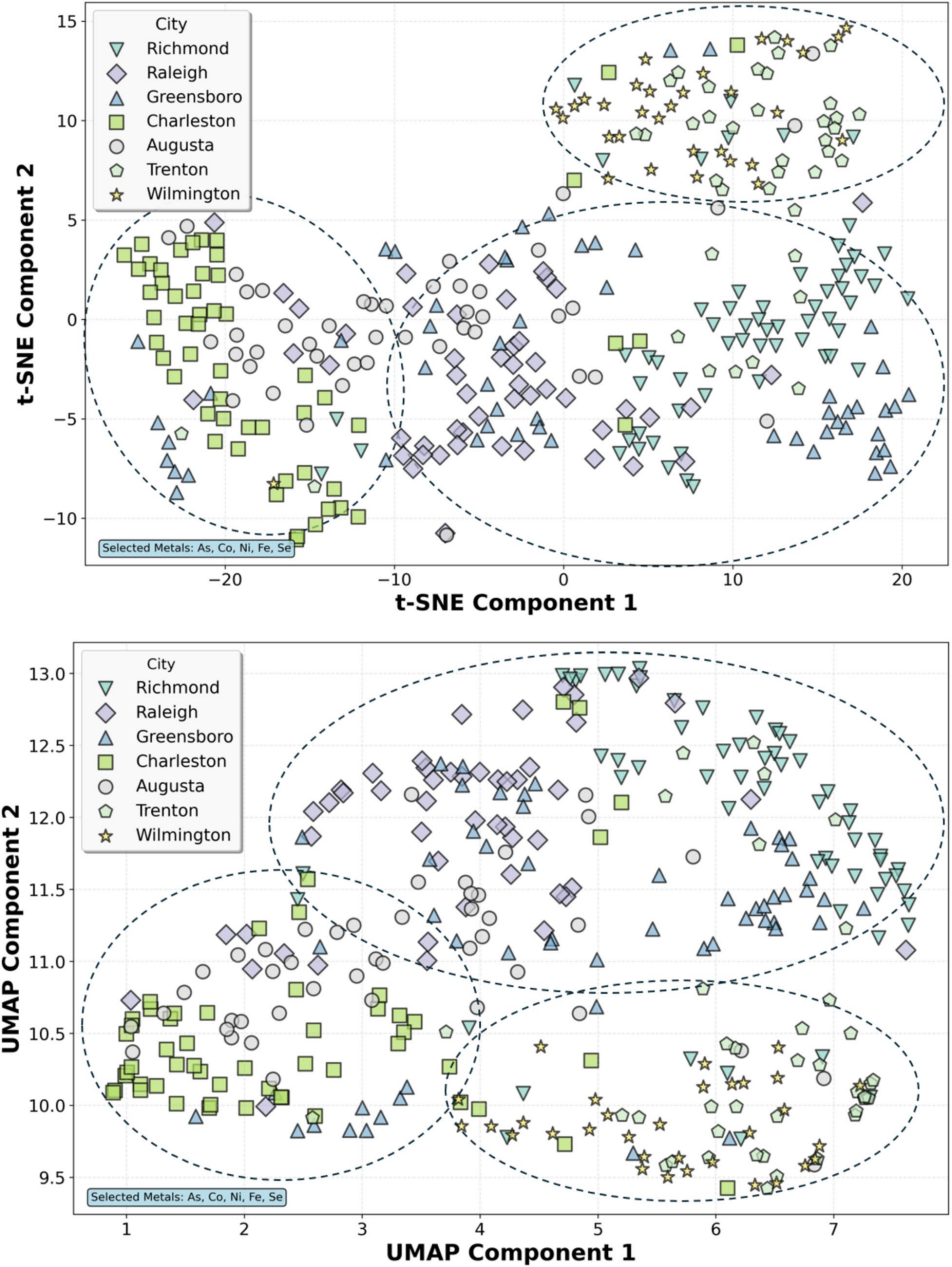
Sample dimensionality reduction and projection using t-SNE (*top*) and UMAP (*bottom*). Data was plotted using top 5 highest discriminatory PTEs for each category

Unlike soil profiles, road dust matrix reflects a higher mixing degree that captures the combined influence of local industrial and anthropogenic inputs. Therefore, our multi-city road dust dataset seeks to highlight the spatial variability in PTEs contamination over the eastern U.S. transect. Figure 2 outlines the t-SNE and UMAP projection result from the chosen set of 11 PTEs (Cu, Zn, As, Se, Ni, Fe, Mo, V, Co, Cd, Pb). All PTEs showed significant differences (Kruskal–Wallis test, *p* < 0.05) among cities, while As, Co, V, Se, and Fe were the top 5 PTEs with highest eta-squared values (η^2^) that highlight the road dust spatial variability (Fig. 2). Enriched As and Se from combustion activities (e.g., waste incinerators, coal combustion), V from port and shipping traffic, Co from industrial abrasion may drive the regional differences in collected samples (Etteieb et al. 2020; Munroe et al. 2025). Northeast riverside industrial cities are known for their historical industrial development and pollution legacy, while Mid-Atlantic Piedmont pollution scenarios are driven by coal-fired power plants and industrial combustion. Southeastern port cities, on the other hand, are logistics hubs with prominent shipping traffic and industrial-freight corridor activities (Hooler et al. 2025**)**. The outlined spatial variability in city development was also reflected in t-SNE/UMAP projection result, with three observed clusters (1) Trenton, Wilmington (northeastern); (2) Richmond, Greensboro, Raleigh (Piedmont); and (3) Charleston, Augusta (southeastern). Also, similar projection results between t-SNE and UMAP analysis revealed a robust and consistent dataset structure, suggesting the observed clusters are inherent local structures that are reproducible with different projection methods. These statistical tools are useful in revealing local clustering patterns in collected road dust, whose variability is influenced by the spatial heterogeneity across sampling locations.

Besides the spatial variability, PTEs composition shows a similar magnitude between cities, with declining sequence as Fe (0.7–2.4%) > Zn (91–178 mg kg^−1^) > Cu (28–116 mg kg^−1^) > Pb (17–60 mg kg^−1^) > V (11–34 mg kg^−1^) > Ni (5–36 mg kg^−1^) > Co (1.5–10.8 mg kg^−1^) > As (1.3–4.2 mg kg^−1^) > Se (1.4–2.7 mg kg^−1^) > Mo (0.8–3.4 mg kg^−1^) > Cd (0.14–0.56 mg kg^−1^, Table 1). Zn, Cu, and Pb were the most prevalent trace PTEs in road dust, indicating the likelihood of atmospheric input from anthropogenic and industrial activities (Dietrich et al. 2022; Fussel et al. 2022; Rehman et al. 2025). Between cities, the northeastern cities (e.g., Trenton, Wilmington) saw generally higher hazardous PTEs concentration (e.g., Pb, As, Cd, Kruskal–Wallis test, *p* < 0.05) than other urban areas. This spatial difference corresponds with the history of anthropogenic and industrial emission profiles along eastern U.S. (i.e., Superfund site distribution, Etteieb et al. 2020; Kiaghadi et al. 2021). Zinc smelting, lead-based pottery, and coal-fired power plants are typical industry sectors that could drive the spatial heterogeneity (Bleiwas and DiFrancesco 2010; Obeng-Gyasi et al. 2021; USEPA, 2025). Despite lower overall PTEs concentrations, early road dust dataset from southeastern cities seek to complement the geographical road dust data gap in the conterminous USA.

**Table 1:** Reported PTE concentrations in surveyed cities (in mg kg^−1^, arithmetic mean ± S.E.) in this study and comparison with other sites

Site	Cu	Zn	As	Se	Ni	Fe	Mo	V	Co	Cd	Pb	Source
Trenton, NJ	116 ± 34	176 ± 20	4.2 ± 0.4	1.9 ± 0.1	18 ± 3	24,713 ± 1427	1.9 ± 0.2	34 ± 3	7.5 ± 0.5	0.28 ± 0.05	34 ± 8	This study
Wilmington, DE	65 ± 8	188 ± 20	3.1 ± 0.4	1.4 ± 0.1	36 ± 15	19,268 ± 1577	3.4 ± 0.6	26 ± 1	7.3 ± 1.6	0.34 ± 0.04	42 ± 7	This study
Richmond, VA	42 ± 6	172 ± 25	4.2 ± 0.3	2.7 ± 0.1	14 ± 1	12,455 ± 523	1.7 ± 0.1	25 ± 1	8.0 ± 1.1	0.56 ± 0.10	60 ± 8	This study
Raleigh, NC	36 ± 8	99 ± 8	1.7 ± 0.4	2.7 ± 0.1	7 ± 1	9706 ± 510	0.9 ± 0.1	24 ± 1	4.6 ± 0.3	0.14 ± 0.02	31 ± 4	This study
Greensboro, NC	56 ± 7	160 ± 19	1.5 ± 0.1	2.6 ± 0.2	11 ± 1	16,140 ± 1395	1.8 ± 0.3	33 ± 3	10.8 ± 0.9	0.19 ± 0.03	44 ± 9	This study
Charleston,SC	28 ± 7	91 ± 13	1.4 ± 0.1	1.4 ± 0.1	5 ± 1	7079 ± 784	0.8 ± 0.1	11 ± 1	1.5 ± 0.1	0.14 ± 0.02	17 ± 3	This study
Augusta, GA	37 ± 12	101 ± 17	1.3 ± 0.2	1.9 ± 0.1	7 ± 1	11,160 ± 1019	1.6 ± 0.5	21 ± 1	3.5 ± 0.4	0.27 ± 0.14	31 ± 11	This study
La Paz, Baja California Sur, Mexico	24.09	132.37	12.98		23.78			112.48	11.80	0.31	38.23	Schiavo et al. (2026)
Fishtown, PA	242.3	713.5			33.27	34,120		65.45	7.45	< 1.5	287.35	O’Shea et al. (2021)
Detroit, MI	102.6	577.3	8.0		40.6	49,350				1.09	134	Denny et al. (2022)
Houston, TX	382	442			169	23,000		16	6.9		89	Fiala and Hwang, (2021)
Salt Lake City, UT and Provo, UT	105	538	11.3		26	27,953	1.71	61	9.2	0.91	108	Munroe et al. (2025)
Havana, Cuba	73.6	548.7			60.6	12,000			7.0		60.8	Díaz Rizo et al. (2023)
Toronto, Canada	121	419	4.2		36		8.4	41	8.0	0.35	63	Wiseman et al. (2021)
Krakow, Poland	573	2690	18.6	261	67.9	59,300	12.5		21.3	5.03	311	Adamiec & Jarosz-Krzemińska (2019)
Opole, Poland	347	1330	6.02	265	116	54,200	9.49		30.2	1.95	216	
Wroclaw, Poland	448	2140	7.50	221	206	59,400	10.8		32.0	6.54	193	
Warszawa, Poland	831	1990	6.56	278	93.4	81,800	22.4		22.2	1.32	216	
Moscow, Russia	66	197	2.0		23	23,831	2.2	53	6.1	0.244	51	Vlasov et al. (2021)
Brussels, Belgium	210.74	353.55			47.83					2.66	117.39	Bogaert et al. (2023)
Bragança, Portugal	4611	2466		0.61	166	95,000		56.4	15.4	3.87	113	Cipoli et al. (2024)

#### Road dust HMs comparison with other studies

On a regional scale, reported road dust PTEs concentrations in our study are lower than other industrial cities and states (e.g., Fishtown, PA–O’Shea et al. 2021; Detroit, MI–Denny et al. 2022; Krakow, Poland–Adamiec and Jarosz-Krzemińska 2019**, **Table 1). However, they are similar to those of Salt Lake City, UT (Munroe et al. 2025); Houston, TX (Fiala and Hwang 2021) and other regional studies in Moscow, Russia (Vlasov et al. 2021); and Toronto, Canada (Wiseman et al. 2021). Road dust Cu and Zn concentrations exceeded WHO permissible limits (36 and 50 mg kg^−1^); while observed As concentrations exceeded U.S. EPA soil screening threshold (0.4 mg kg^−1^, WHO, 1996; USEPA, 1996). The observed exceedance of regulated level surpassed the neutralizing capacity from urban soil and vegetation, therefore heightening the risk of adverse health outcomes. Furthermore, road dust data in North America and Europe is relatively scarcer, necessitating more data reporting using ICP-OES/ICP-MS techniques. This will also overcome the lack of analytical precision from handheld p-XRF data and allowing for accurate cross-study comparative assessment (Fussell et al. 2022; Tong et al. 2023; Roy et al. 2024).

#### Enrichment factor assessment

**Fig. 3:**
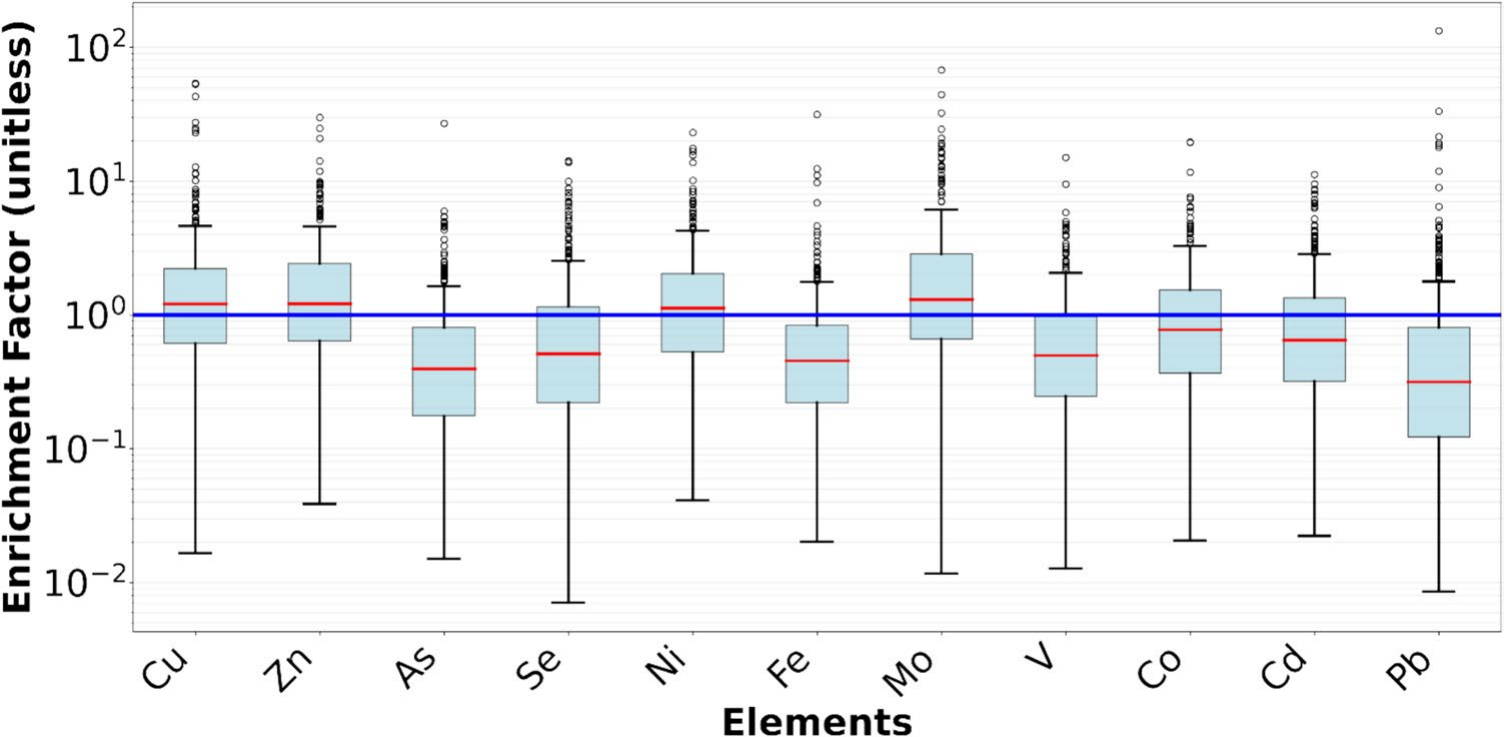
Enrichment factor (EF) box and whisker plots of selected PTEs in overall case (blue line shows EF = 1)

In addition to concentration, enrichment factors can help discern the PTEs with non-lithogenic accumulation from industrial and anthropogenic sources, using Sr as the leveraging agent (Fig. 3, Figure S3). Significant enrichment (EF > 1) was found for Cu, Zn, Mo, and Ni for the overall case while there was minimal enrichment (EF < 1) for hazardous metals (e.g., Pb, As, Cd). Possible enrichment effects from modern pollution sources such as non-exhaust traffic emission, industrial activity (e.g., mining, smelting) could be responsible for elevated PTEs level. Hazardous elements, on the other hand, tend to share a similar magnitude with those of subsurface soil (10–20 cm depth). This implies the influence of legacy contamination in urbanized areas, stemming from historical input of coal combustion, pesticide application, and waste incineration (Obeng-Gyasi et al. 2021; Filippelli et al. 2024). However, outlier EF values of surveyed PTEs fell into categories of moderate (5 ≤ EF < 10) and extreme enrichment ratios (EF ≥ 50, Fig. 3). These “hotspots” possesses higher proportions of modern pollution sources, with higher bioavailability and more toxic influence towards local residents upon exposure. On the other hand, EF calculations using Al (a conventional normalizer) generally yielded 3.5-time higher values than those of Sr (Figure S4). However, the same enrichment pattern was observed, with higher EFs observed for modern pollution sources (Cu, Zn, Mo, Ni) than hazardous legacy metals (Pb, As). In urban settings, Fe and Al have anthropogenic inputs from manufacturing industry, steel production, vehicle wear, and tear etc., that strongly influence their background roles (Howard et al. 2013; Vlasov et al. 2022). Therefore, the choice of Sr as a “preservative” element allowed for more accurate EF assessment, since Sr strictly tracks crustal mineral weathering with limited isotope fractionation and could be applied as the lithogenic background **(**Salifu et al. 2018; Kebonye and Eze 2019). In our study, road dust and subsurface soil Sr concentrations range from 8.1 to 46.0 mg kg^−1^ and 2.7–23.3 mg kg^−1^ (Table S4), matching labile soil Sr concentration background (0.2–20 mg kg^−1^, ATSDR, 2004) needed for EF calculation.

### Road dust PTEs hotspot and potential impact

**Fig. 4:**
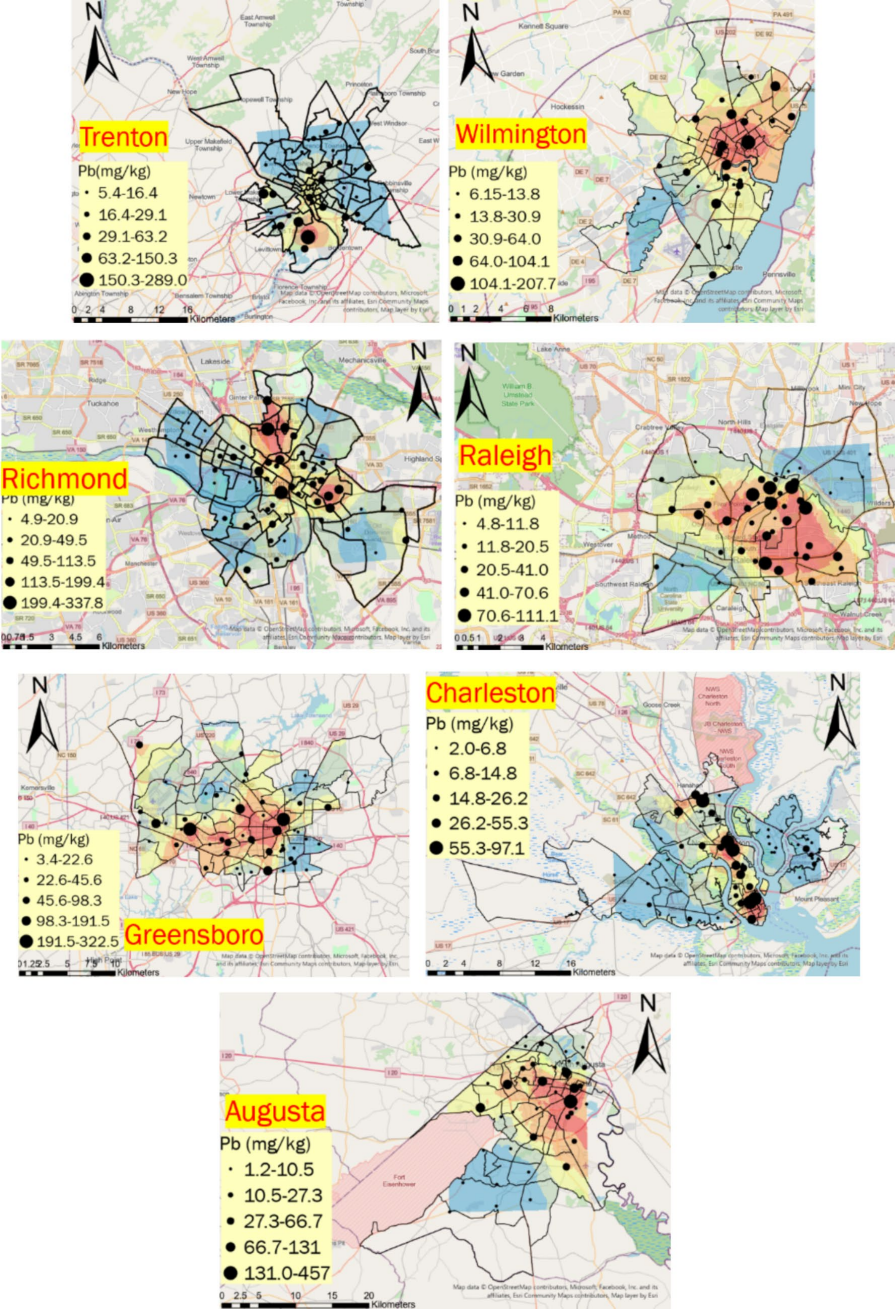
Spatial kriging analysis (EBK) for road dust Pb concentration at surveyed cities

Besides the inter-variability (i.e., between-city) in PTEs concentration, geospatial analysis is further integrated in each city to reveal the intra-variability (i.e., within-city) of typical PTEs. Lead (Pb) has been extensively studied as a prominent, urban legacy contaminant to residents, with recent U.S. EPA revised Pb guidance level of 200 mg kg^−1^ for residential soil and 5 ug dL^−1^ for blood level (Dietrich et al. 2022; Obeng-Gyasi et al. 2021). Figure 4 outlines the spatial road dust Pb pattern in surveyed cities. Pb hotspots exceeding EPA guidance levels are found in downtown areas except for Raleigh, NC and Charleston, SC; aligning well with previously reported low Pb soil concentration in southeastern states (Pace and Di Giulio 1987; Aelion et al. 2008, USEPA 2025). However, the observed Pb spatial distribution did not closely overlap with those traffic-related PTE markers (Cu and Zn, Figure S1 and S2), which shows higher concentrations in suburban areas. This pattern shows the potential Pb input from other sources than traffic, potentially from lead-based paint, smelters, and coal-fired power plant (Wade et al. 2021**; **Wang et al. 2022; Filippeli et al. 2024).

**Fig. 5:**
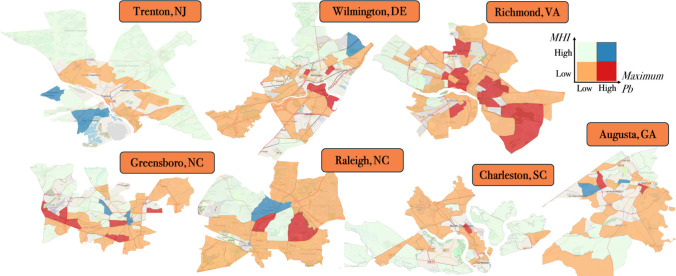
Bivariate display of household income and maximum road dust Pb concentration at census-tract level. (Low income defined as income < 80% area median income, city-specific but approx. $100,000, high Pb defined as tracts with Pb concentration > 80 mg kg^−1^ per CA soil guidelines, Branch 2009; Begley et al. 2021)

To further visualize the potential impact of road dust Pb pollution, Fig. 5 seeks to overlay road dust Pb concentration with 2020 U.S. Census Tract MHI data. California soil Pb regulation level (80 mg kg^−1^) was set as the flagging criterion, while low-income is set at MHI < 80% of the area median income for each city (Table S3, between $70,000 and $101,000). It can be seen that low-income census tracts are more susceptible with high Pb exposure (Fig. 5, *in red*), while high-income neighborhoods in suburban periphery observed lower Pb exposure (*in green*). The observed spatial heterogeneity in Pb risk highlighted a potential imbalance in pollution exposure across eastern U.S. municipalities—where elevated pollution stemming from historical urban development could disproportionately impact certain demographics. Other PTEs from other activities (i.e., Cu, Zn from traffic) might not exhibit a similar pattern, and additional analyses in future studies are needed to address potential causal relationships between PTEs concentration and other socioeconomic variables.

### Urban PTEs sources apportionment and quantification

#### Correlation and biplot PCA analysis

**Fig. 6:**
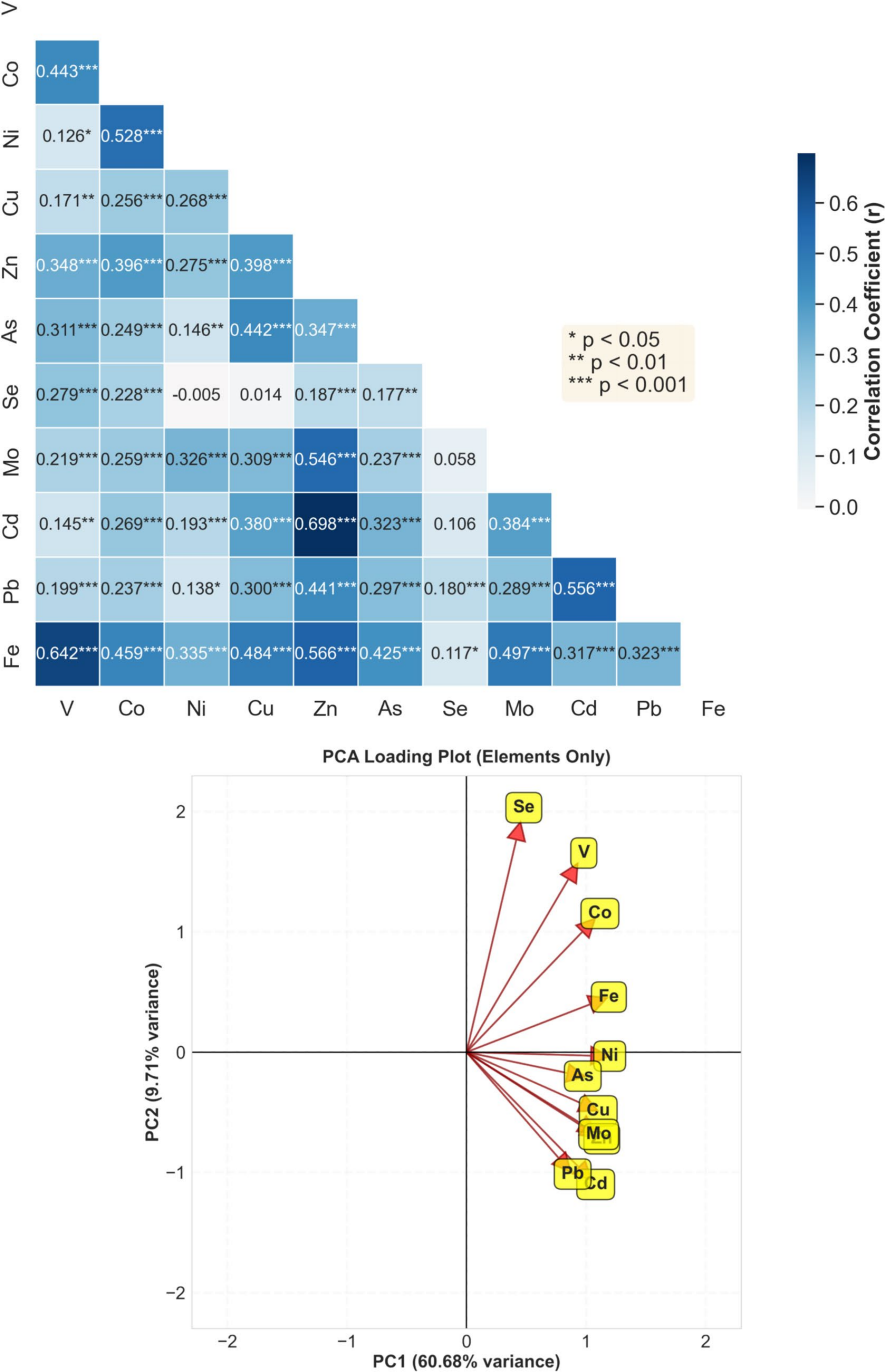
Pearson’s correlation analysis (top) and PCA biplot (bottom) for PTEs in this study

In the context of urban pollution, the co-emission of PTEs derived from similar origins provides critical insights into primary road dust sources. Pearson’s correlation matrix in Fig. 6 highlights the relationship between PTEs in this study. Generally, the strongest correlations of Fe-V (*r* = *0.*64), and Zn-Cd (*r* = 0.70) might suggest the inputs from industrial emission, heavy oil fuel, and waste incineration in surveyed cities (Thorstenson et al. 2025). Cobalt (Co) demonstrated strongest correlation (*r* = 0.53) with Ni compared to other PTEs, hinting the contribution of electronic waste from manufacturing and landfills (Pitawala et al. 2025). Copper (Cu) and zinc (Zn) serve as the non-exhaust traffic emission (e.g., tire wear, break wear) for urbanized area and show a moderate correlation (*r* = 0.40, Fussell et al. 2022**; **Liu et al. 2023). Nonetheless, almost all chosen PTEs exhibited significant, positive correlation (*r* > 0, *p* < 0.05), indicating their susceptibility for assessment by positive matrix factorization (PMF) in the next section.

Beyond correlations, between-elements PTEs variability was also captured by the first two principal components (PCs) in PCA biplot analysis, which explained 70.4% total variance (Fig. 6). On a distance basis, selenium (Se) stood out on the upper right quadrant as an individual source on PC1. Hazardous PTEs (e.g., Pb, As, Cd) were seen on the lower right quadrant as another group of sources on PC2. The greatest statistical distance was observed between Pb and Se, suggesting distinct geochemical behaviors in collected road dust samples. Among the 5 most discriminatory PTEs shown in t-SNE/UMAP analysis (As, Co, Ni, Fe, Se), only arsenic (As) occupied a distinct quadrant. Observed moderate Pb-As correlation (*r* = 0.30) pinpoints toward arsenic-based insecticides, a practice heavily utilized in historical agricultural regions nested in the Cotton Belt (e.g., Charleston, SC; Augusta, GA; Bednar et al. 2002). The clustering of other PTEs (Co, Ni, Fe, Se) in PC1 further outlines their likelihood to be contributed from industrial and combustion processes, two common contemporary sources that have been extensively referred to by other studies (Kasongo et al. 2024; Roy et al. 2024). 

#### PMF source apportionment result

**Fig. 7:**
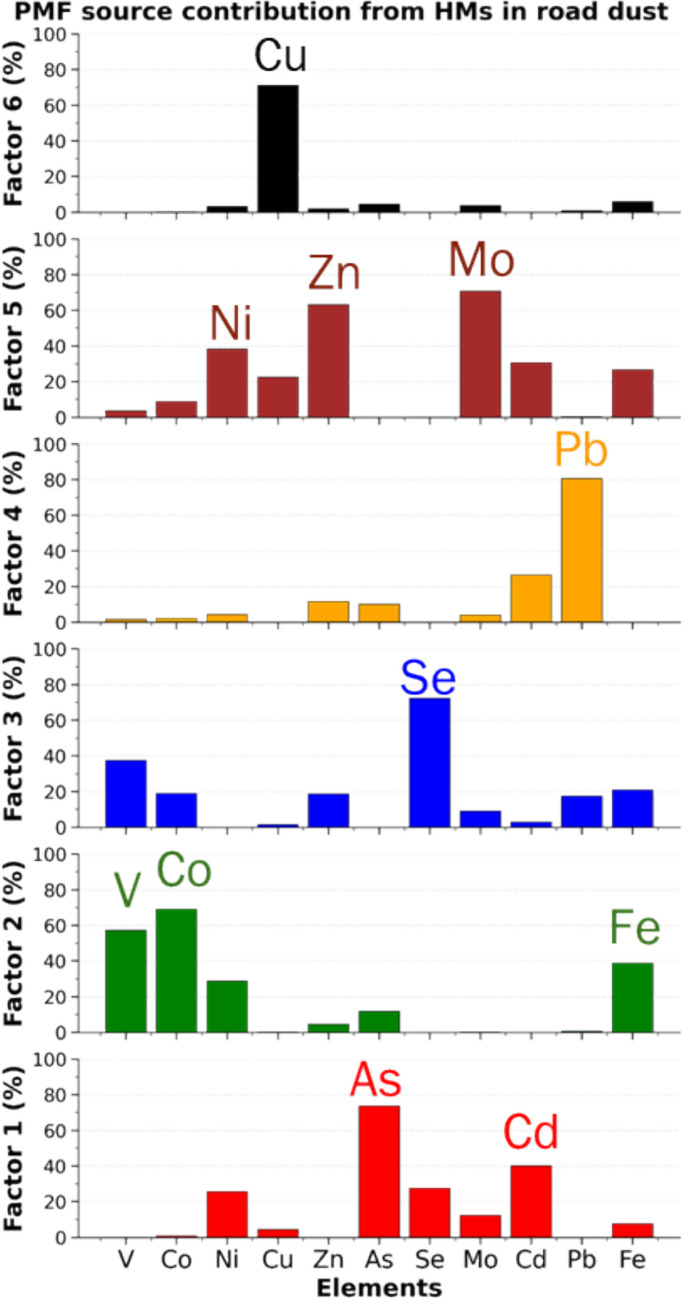
PMF analysis result for collective road dust samples at surveyed cities in this study

Furthermore, PMF serves as a mathematical receptor model for accurately quantifying the contribution of each pollution group outlined by PCA and correlation analysis. Table 2 and Fig. 7 outline the 6-factor PMF solution derived from collected road dust in this study. Se (18.1%, Factor 3); Pb (12.9%, Factor 4); and Cu (8.3%, Factor 6) stood out as individual factors from selected solution. Selenium has long been considered the primary tracer for coal combustion, either from coal-fired power plants or direct combustion with historical operation in central eastern U.S. (e.g., VA, NC, SC; Etteieb et al. 2020). As outlined by previous sections, non-exhaust traffic emission and coal-fired power plants/pesticide use might explain Cu and Pb variability in our samples, respectively. The presence of waste incinerators in urbanized areas, on the other hand, are known inputs for Cd while also serving as another As source (17.1%, Factor 1, Kim and Lee 2017). Charleston, SC and Raleigh, NC are the two cities that opted for landfill over incineration, hence seeing lower As and Cd concentration than others (Kruskal–Wallis test, *p* < 0.05, Table 1). Other localized sources include Ni, Zn, Mo (24.1%, Factor 5) and V, Co, Fe (heavy fuel combustion, 19.2%, Factor 2). The common use of heavy fuel combustion in southeastern port U.S. cities and industrial-freight activities explain the presence of PTEs in factor 2. Molybdenum (Mo) and nickel (Ni) are typical PTEs seen in manufacturing, steel production, refineries etc., that contributes to factor 3 (Roy et al. 2024; Pitawala et al. 2025). Enrichment of road dust Mo at Wilmington, DE is attributable to local metal coating industry, explaining its highest Mo bulk concentration and its origin in our PMF solution. Overall, hazardous PTEs (e.g., Pb, As, Cd) contributed one-third (31%) to the road dust pollution sources in eastern U.S. municipalities, mostly originating from coal combustion, coal-fired power plant, and insecticide use. Other PTEs accounted for the other two-thirds (69%), covering non-exhaust traffic emission, industrial activity, and maritime port activity. However, information from pseudo-total digestion cannot confirm the existence of wear particles in our samples, whereas microscopic imaging equipped with X-ray microanalysis (SEM–EDS) in future studies would offer a more reliable complementary approach. Nonetheless, these identified sources will provide an outline to evaluate ancillary environmental matrices and facilitate comprehensive assessment of legacy sources (e.g., lead legacy, coal-fired power plants)—which could exhibit elevated exposure risks towards certain demographics.

**Table 2:** 6-factor PMF solutions derived from road dust samples collected in all surveyed cities

Factor	%Contribution	Element	Sources
1	17.5	As, Cd	Waste incinerator, insecticide use
2	19.2	V, Co, Fe	Port activity (heavy oil combustion, industrial-freight corridors)
3	18.1	Se	Coal combustion
4	12.9	Pb	Coal-fired power plants, insecticide use
5	24.1	Ni, Zn, Mo	Industrial manufacturing, metal coating
6	8.3	Cu	Non-exhaust vehicle emission

### Health risk assessment

**Fig. 8:**
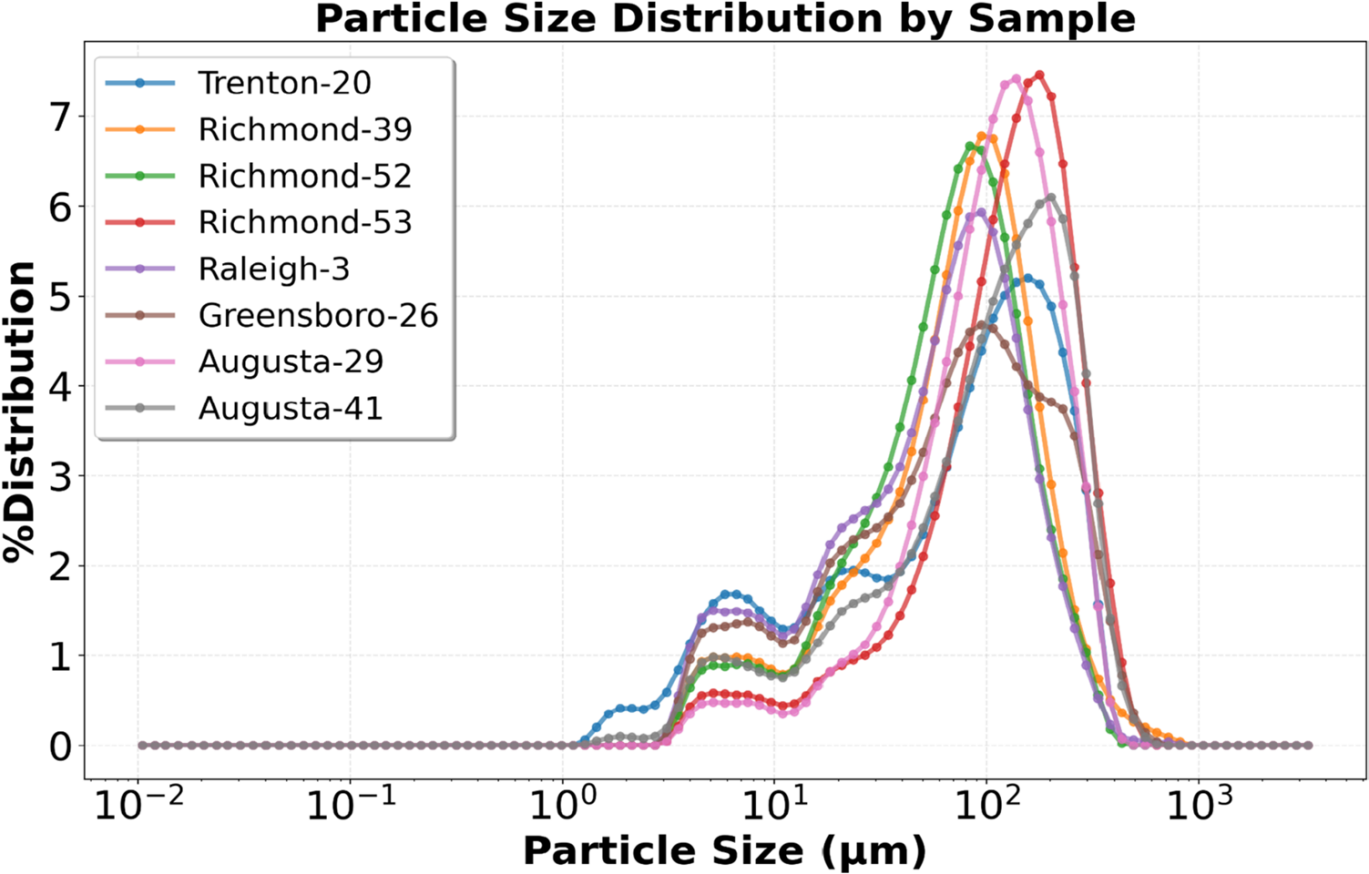
Grain size distribution of samples with high concentration of toxic PTEs (Pb, As, Cd)

**Fig. 9:**
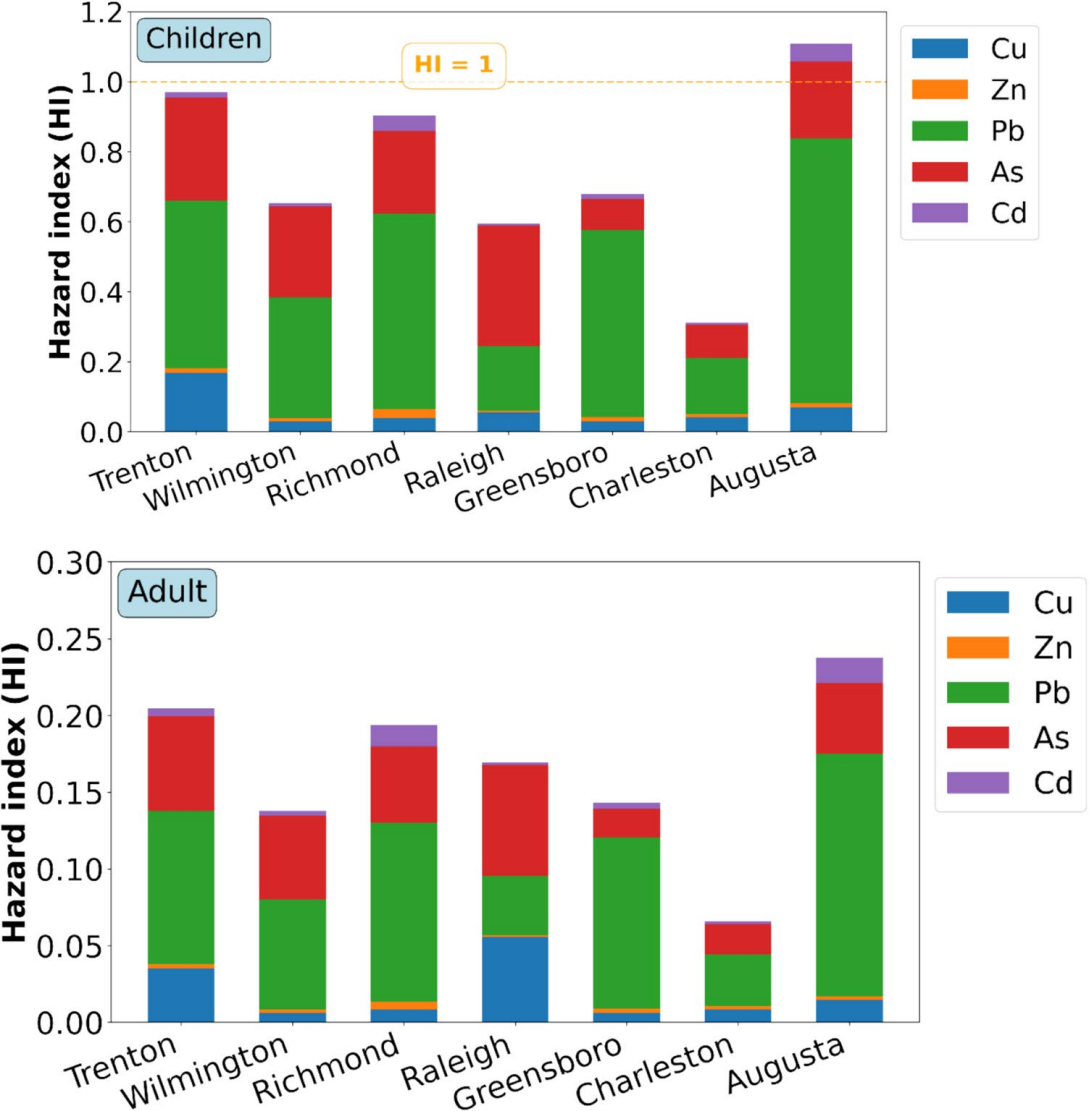
Hazard index (HI) values for maximum PTEs exposure on children and adults in eastern U.S. cities

Road dust pollution could be detrimental to public health owing to the respirable dust fraction (< 10 μm) with higher inhalation risk upon exposure. Respirable dust has longer atmospheric residence time, typically characterized as particulate matter (PM) with lower deposition rates and could enter the pulmonary system directly upon inhalation, leading to greater exposure (Wippich et al. 2020; Alijagic et al 2024). Figure 8 shows the grain size distribution for road dust samples exceeding the EPA soil screening guidelines for Pb and As (at 200 mg kg^−1^ and 0.68 mg kg^−1^, respectively). A bimodal (or trimodal) grain size distribution was observed for collected road dust, with notable modes at 7.0–8.0 μm, 15.0–17.1 μm, and 69–78 μm. Nonetheless, higher proportion at the finer mode (up to 10%, at 7.0–8.0 μm, Fig. 9) was observed for Trenton, Raleigh, and Greensboro. This emphasized the spatial heterogeneity in road dust PTEs exposure, in which our findings are likely to provide the lower-bound estimate of the true ecotoxicology impact on urban residents.

Nonetheless, an attempt to estimate the potential risks from five typical PTEs (e.g., Cu, Zn, Pb, As, Cd) is shown in Fig. 9. Maximum Cu, Zn, Pb, As, and Cd concentrations were chosen to evaluate the impact on children (< 13 years old) and adults (13–65 years old). Lead (Pb) was the primary contributor to the HI for both age groups, followed by As, Zn, Cu, and Cd. This pattern reinforced the findings in the previous sections, highlighting the direct impact of Pb and As sources (i.e., coal-fired power plants and insecticide use) on public health in these areas. Children generally have a 4.5-time higher HI values (0.65–1.11) on average as compared to those of adults (0.07–0.24), calling for different measures for exposure mitigation for different age groups. Among all cities, only Augusta, GA showed a cumulative HI > 1 suggesting possible adverse health risk, but these values encourage additional data collection in southeastern cities. These HI values gave a better precautionary level than the median PTEs concentration, which showed 4.5–13.1 times lower values than the “maximum concentrations” framework (Table S5) and might not show the possible “true” exposure at hotspot regions. Despite the lack of inter-annual data to capture HI value variation throughout the year, health risk assessment in this study offers potential guidance for estimating dust pollution exposure risk. Seasonal data monitoring and additional age and demographic-specific assessments would be helpful to further quantify potential exposure risk to road dust PTEs in the future. Therefore, HI values derived from median concentration are still strongly suggested for comparison purposes, as median road dust PTEs concentration could be higher in other regions (Table 1).

## Conclusion

In this study, concentrations of 11 PTEs (Cu, Zn, As, Se, Ni, Fe, Mo, V, Co, Cd, Pb) in road dust were reported in 7 mid-size cities along the eastern U.S. Dimensionality reduction analysis using t-SNE/UMAP outlined a regional difference from PTEs spatial variability, revealed by As, Co, V, Se, and Fe. This pattern matched the industrial profiles of eastern U.S., with northeastern historical industrial centers (Trenton, Wilmington), Piedmont coal-fired power plants and coal combustion (Richmond, Raleigh, Greensboro), and southeastern maritime port activities (Charleston, Augusta). Despite showing a typical urban elemental profile, toxic PTEs (Cu, Zn, Pb, As, Cd) exhibited hotspots with higher levels than several soil guidelines from the U.S. EPA and WHO. Concentrations of Cu, Zn, Mo, and Ni showed significant enrichment (EF > 1), while below-unit Pb, As, and Cd EFs proposed the effect from historical combustion, waste incineration, and insecticide uses in these urban regions. Furthermore, hazardous Pb hotspots violating EPA guidelines (> 200 mg kg^−1^) were associated with low-income communities in downtown area, outlining a potential skewed exposure issue. Source apportionment analysis using PCA and PMF also revealed a one-third contribution of hazardous metals (e.g., Pb, As, Cd, 31%) and two-thirds contribution from other PTEs (69%), comprising those from traffic, maritime port activities and industrial activities. Particle grain size analysis further revealed up to 10% of respirable dust fraction (< 10 μm) observed at Trenton, Raleigh, and Greensboro, outlining potentially higher risk from road dust exposure. Cumulative hazard index (HI) assessment from maximum PTEs concentrations (Pb, As, Cd, Cu, Zn) indicated Augusta, GA with possible adverse health risk (HI > 1) from inhalation and ingestion; but children experienced up to 4.5-time higher HI values than adults. This study decoded the complex spatial variability in urban road dust bulk PTEs concentration along the eastern U.S. cities, addressed the data gap in southeastern municipalities, performed source apportionment and a first-order health risk assessment for under-reported cities. The introduced geochemical framework provides a robust approach to identify road dust pollution sources impacting local communities, with strong potential to apply for other understudied regions.

## Supplementary Information

Below is the link to the electronic supplementary material.

Supplementary Material File 1 (DOCX 9.48 MB)

## Data Availability

Data supporting the fi ndings of this study are made available from the authors upon reasonable request.
