# Supplementary material for: Tracing spatial mid-size Eastern U.S. cities road dust pollution: insights from source apportionment and health risk assessment
Source: Environ Sci Pollut Res Int. 2026 Jul 7;33(21):10936–53. doi: 10.1007/s11356-026-38034-x (PMC13369771; doi:10.1007/s11356-026-38034-x)
Supplement: Supplementary file 1 — Supplementary Material File 1 (DOCX 9.48 MB) [file 11356_2026_38034_MOESM1_ESM.docx]

**Supplementary Information for**

 TRACING SPATIAL MID-SIZE EASTERN U.S. CITIES ROAD DUST POLLUTION:

INSIGHTS FROM SOURCE APPORTIONMENT AND HEALTH RISK ASSESSMENT

Minh-Tri Truong^1*†^, Chattan T. Haselden^1†^, Anh-Chi Tuan^2^, Justin B. Richardson^1^

^1^ Department of Environmental Sciences, University of Virginia, Charlottesville, VA USA 22904

^2^ Department of Mathematics, Politecnico di Milano, Milan, Italy

*† These authors contributed equally to this work*

*Corresponding author: Minh Tri Truong (ayd7dt@virginia.edu)

**
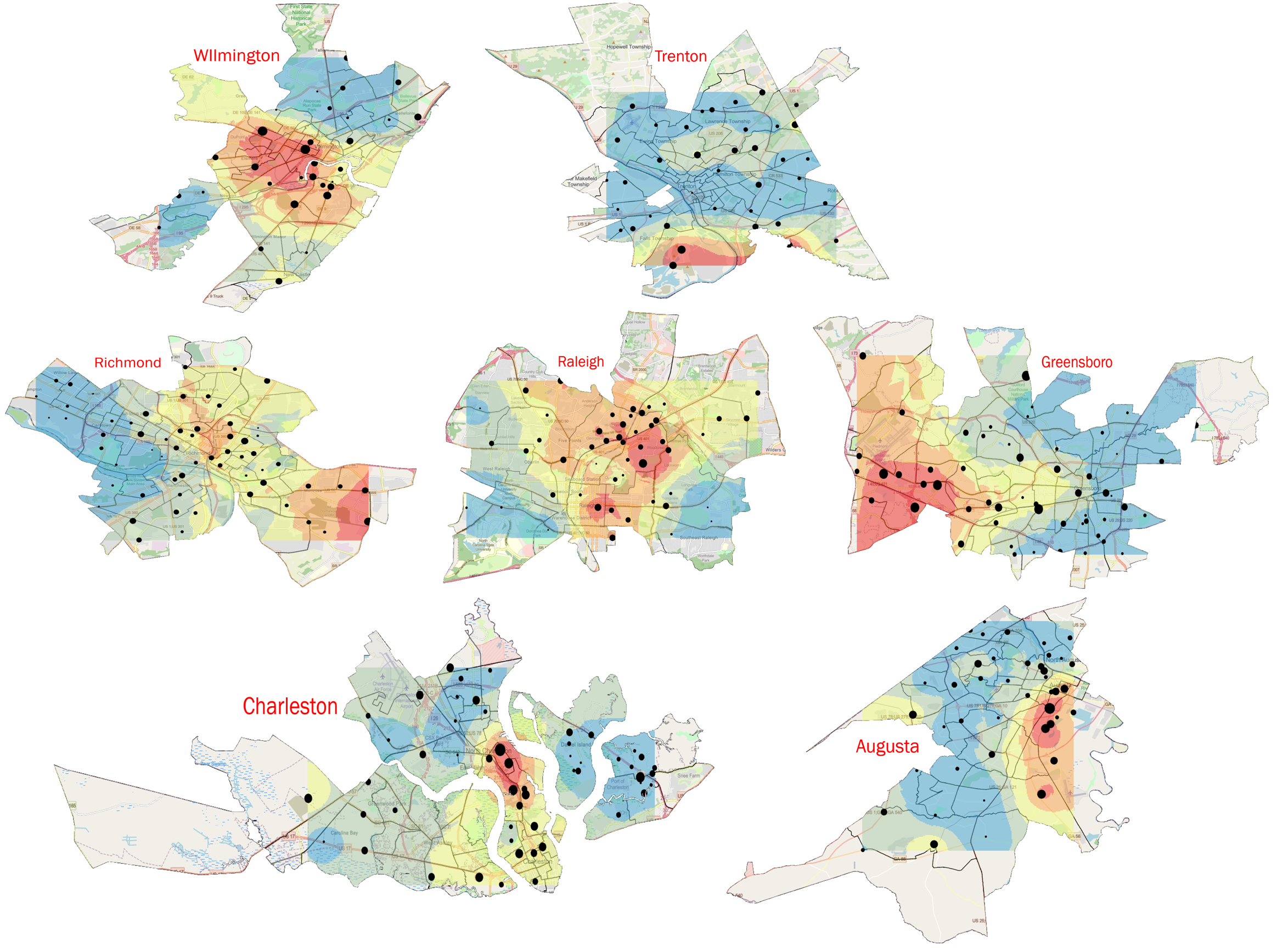
**

**Figure S1.** Empirical Bayesian Kriging (EBK) result for road dust Cu concentration at selected cities


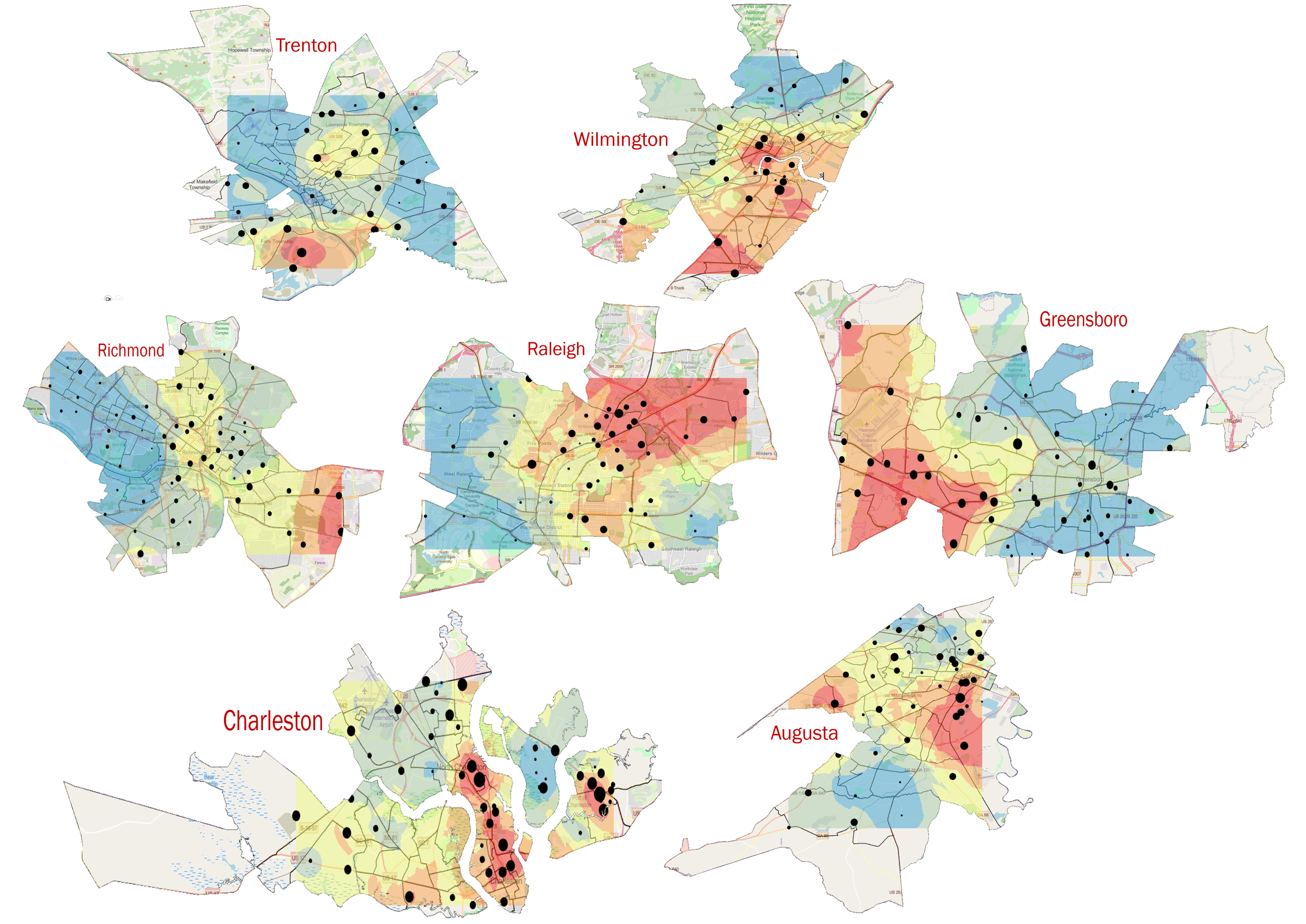


**Figure S2.** Empirical Bayesian Kriging (EBK) result for road dust Zn concentration at selected cities

**
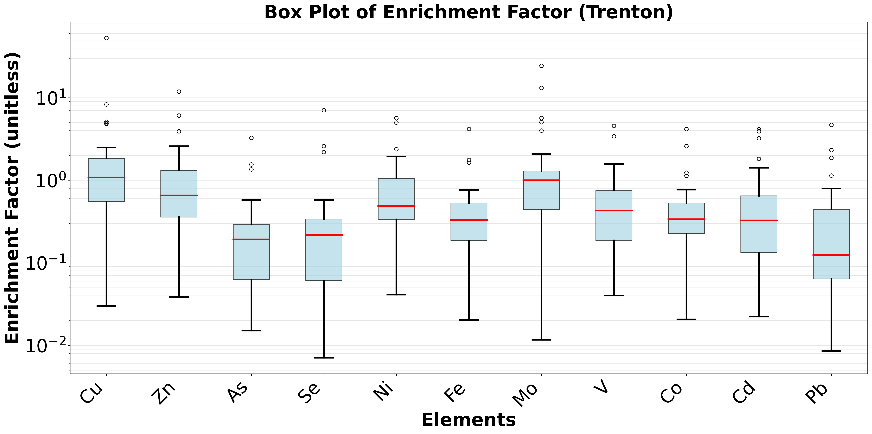
**
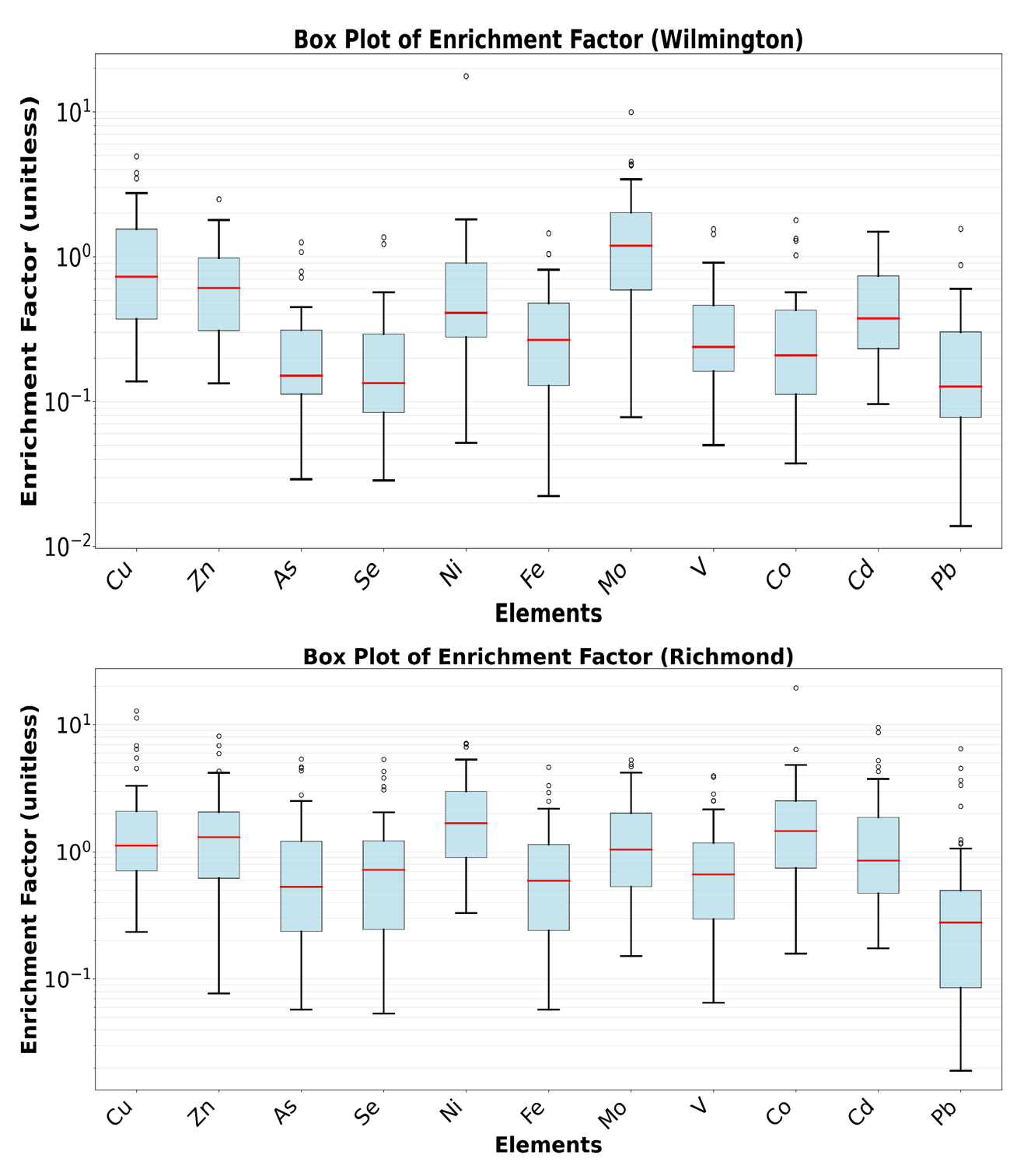


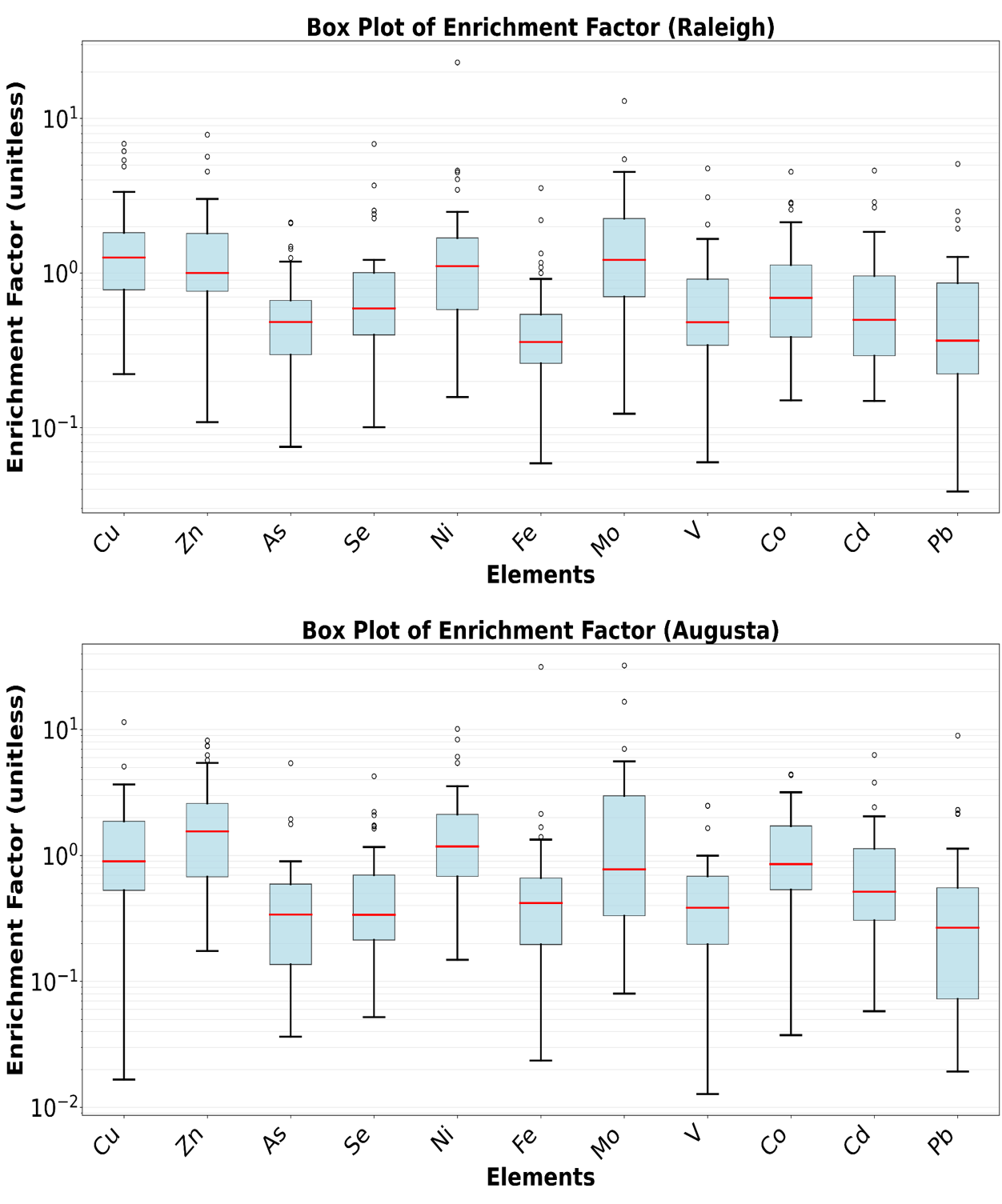

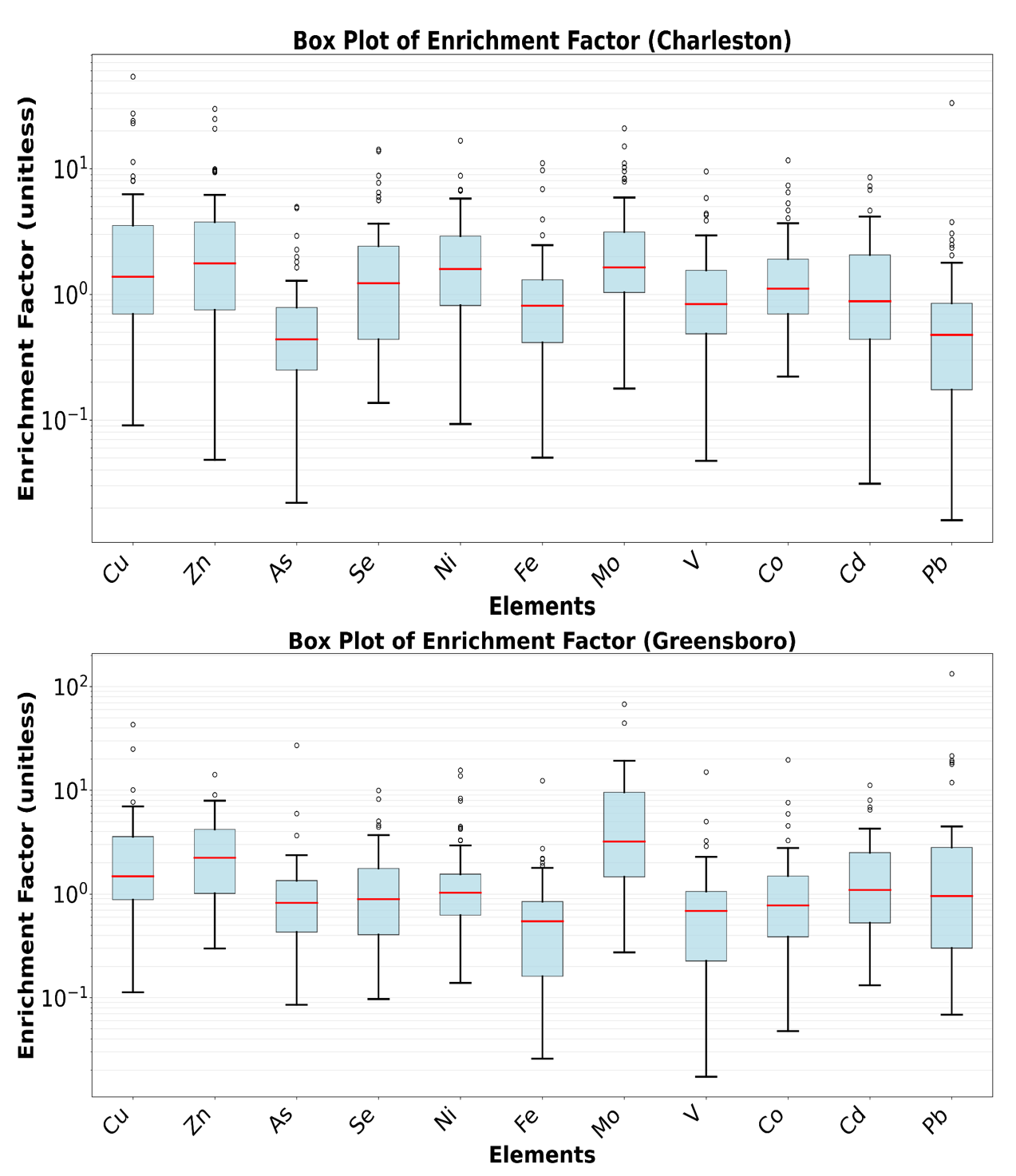


**Figure S3.** Enrichment factor (EF) box and whisker plots of selected PTEs for each city


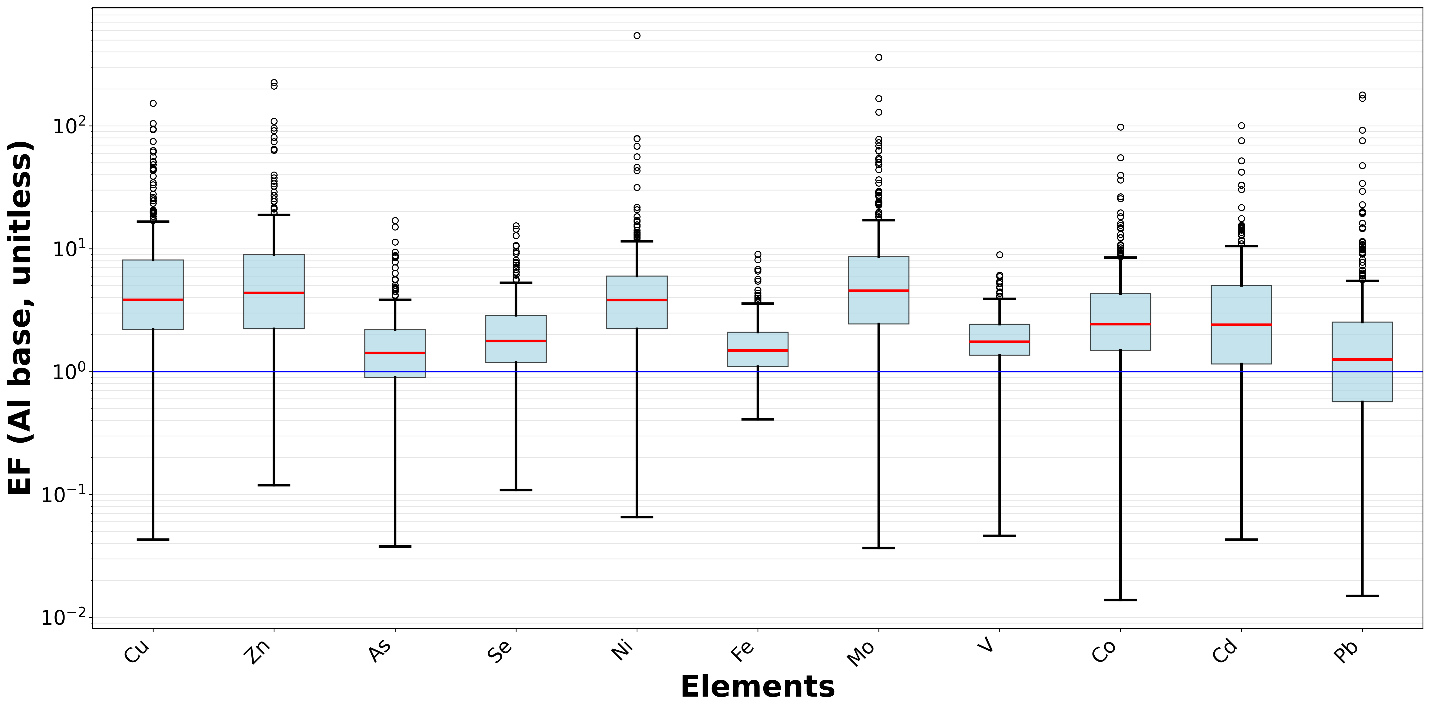


**Figure S4.** Enrichment factor (EFs) box and whisker plots of cases using Aluminum (Al) as normalizer (blue line shows EF = 1)

**
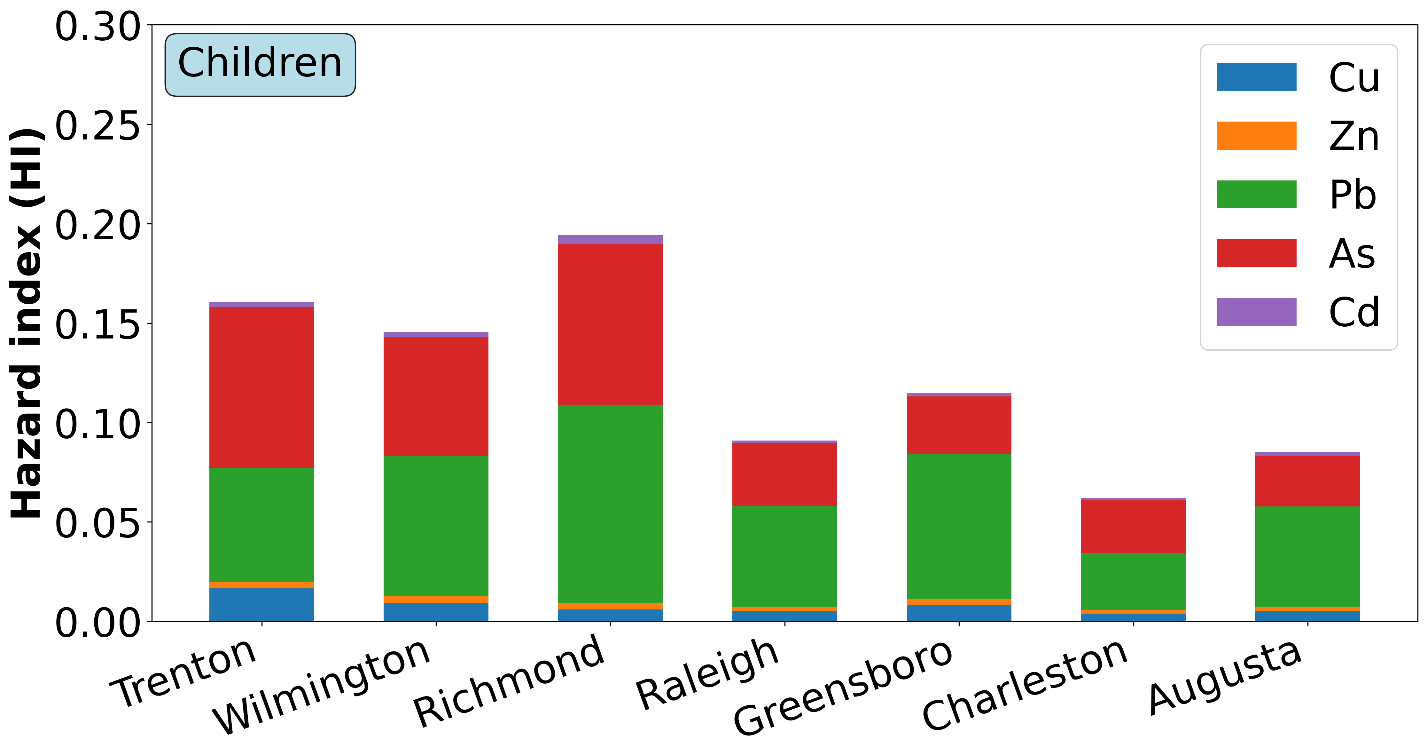
**

**
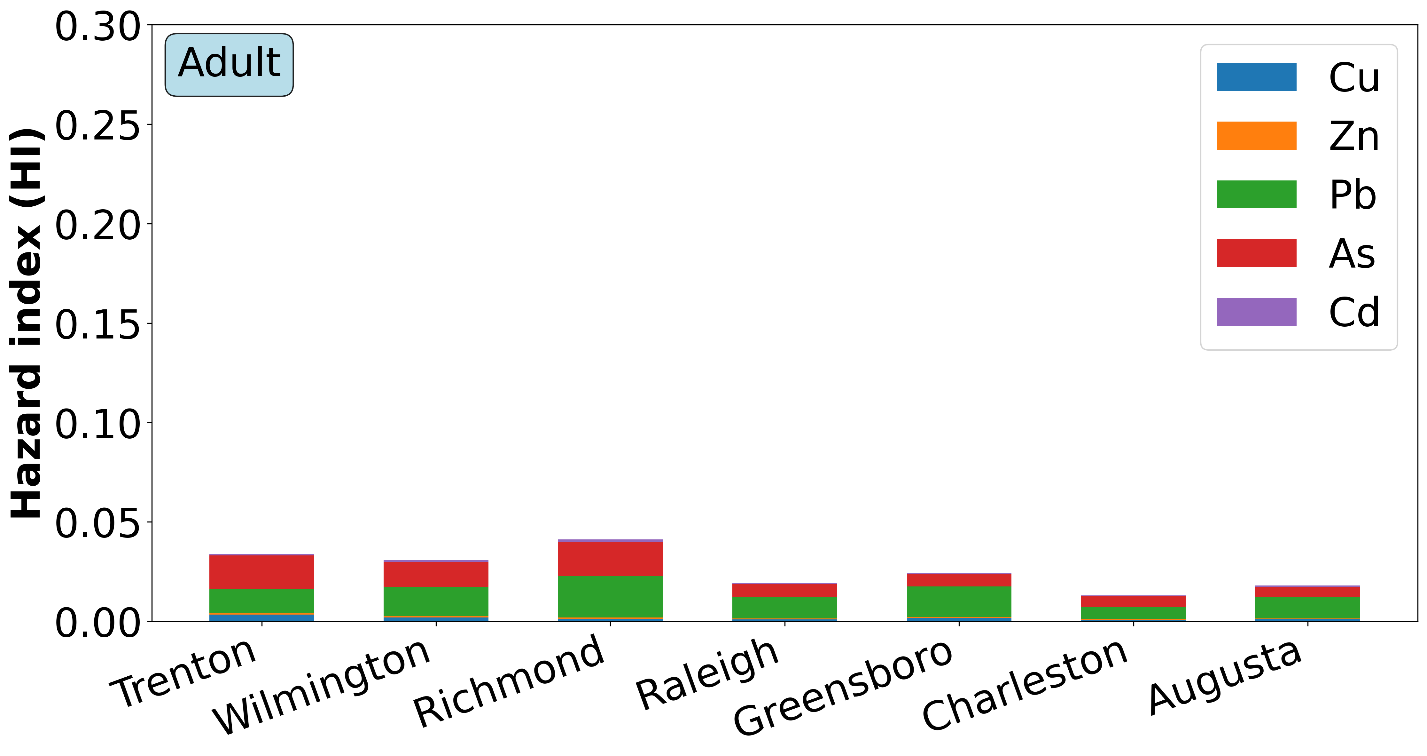
**

**Figure S5.** Hazard index (HI) values for average PTEs exposure on children and adults in eastern U.S. cities

**Table S1**. Recovery rates (Mean ± S.E., in %) from SRM 2710a and SRM 2587 for the 11 chosen PTEs

|  | Cu | Zn | As | Ni | Fe | Co | Cd | Pb |
| --- | --- | --- | --- | --- | --- | --- | --- | --- |
| SRM 2710a  (n = 17) | 105.7 ± 2.5 | 107.8 ± 2.2 | 112.4 ± 5.3 | 105.0 ± 2.9 | 94.1 ± 2.6 | 78.8 ± 3.3 | 104.9 ± 5.9 | 109.0 ± 2.5 |
| SRM 2587  (n = 20) |  |  | 85.3 ± 3.6 |  |  |  | 87.1 ± 2.6 | 112.6 ± 2.7 |

**Table S2.** Empirical Bayesian Kriging (EBK) semivariogram model for HMs concentration interpolation

| Output type | Prediction |
| --- | --- |
| Transformation Type | Log-empirical |
| Semivariogram Model Type | Exponential |
| Subset Size | 100 |
| Overlap Factor | 1 |
| Number of Simulations | 100 |
| Searching neighbourhood | Standard NOSectorsNO Ellipse |
| Max # Neighbours to include | 10 |
| Min # Neighbours to include | 5 |
| Major semi-axis | 0.00241 |

**Table S3.** Area median income used as low-income filter for bivariate analysis (adapted from Area Median Income Lookup Tool (AMI), accessed March 2026)

| **City** | **Low-income limit** |
| --- | --- |
| Trenton, NJ | $ 101,520 |
| Wilmington, DE | $ 95,520 |
| Richmond, VA | $ 90,720 |
| Raleigh, NC | $ 106,160 |
| Greensboro, NC | $ 68,160 |
| Charleston, SC | $ 88,720 |
| Augusta, GA | $ 70,560 |

**Table S4.** Strontium concentration (Sr, in mg kg^-1^) in road dust and subsurface soil used for enrichment factor (EF) calculation in this study

| Sr (Average ± SE) | Trenton | Wilmington | Richmond | Raleigh | Greensboro | Charleston | Augusta |
| --- | --- | --- | --- | --- | --- | --- | --- |
| Road dust | 46.0 ± 6.8 | 37.4 ± 4.6 | 13.2 ± 1.0 | 9.6 ± 1.0 | 15.0 ± 1.8 | 19.9 ± 2.5 | 8.1 ± 1.0 |
| Subsurface soil | 17.3 ± 7.0 | 10.4 ± 1.4 | 8.3 ± 0.9 | 6.5 ± 1.0 | 12.4 ± 1.7 | 23.3 ± 6.6 | 2.7 ± 0.4 |
